# Role of Peroxisome Proliferator-Activated Receptor Alpha in the Control of Cyclooxygenase 2 and Vascular Endothelial Growth Factor: Involvement in Tumor Growth

**DOI:** 10.1155/2008/352437

**Published:** 2008-07-28

**Authors:** Raquel Grau, Manuel D. Díaz-Muñoz, Cristina Cacheiro-Llaguno, Manuel Fresno, Miguel A. Iñiguez

**Affiliations:** Departamento de Biología Molecular, Centro de Biología Molecular “Severo Ochoa” UAM-CSIC, Universidad Autónoma de Madrid, 28049 Madrid, Spain

## Abstract

A growing body of evidence indicates that PPAR (peroxisome proliferator-activated receptor) *α* agonists might have therapeutic usefulness in antitumoral therapy by decreasing abnormal cell growth, and reducing tumoral angiogenesis. Most of the anti-inflammatory and antineoplastic properties of PPAR ligands are due to their inhibitory effects on transcription of a variety of genes involved in inflammation, cell growth and angiogenesis. Cyclooxygenase (COX)-2 and vascular endothelial growth factor (VEGF) are crucial agents in inflammatory and angiogenic processes. They also have been significantly associated to cell proliferation, tumor growth, and metastasis, promoting tumor-associated angiogenesis. Aberrant expression of VEGF and COX-2 has been observed in a variety of tumors, pointing to these proteins as important therapeutic targets in the treatment of pathological angiogenesis and tumor growth. This review summarizes the current understanding of the role of PPAR*α* and its ligands in the regulation of COX-2 and VEGF gene expression in the context of tumor progression.

## 1. INTRODUCTION

Peroxisome proliferator-activated receptors (PPARs) are members of the nuclear receptor family of transcription factors. They modulate gene transcription in response to specific ligands by binding as heterodimers with the retinoid X receptor (RXR) to a specific peroxisome proliferator-response element (PPREs) on target genes (reviewed in [1, 2]). Three distinct isoforms of PPARs have been described PPAR *α*, *β*/*δ*, and *γ*, each encoded by a different gene and showing a distinct tissue distribution [3]. Originally found to be involved in the regulation of lipid and glucose metabolism, [4, 5] interest in these receptors has increased dramatically as a consequence of recent studies showing their involvement in tumoral cell growth and inflammation (reviewed in [6–9]). Therefore, in addition to their well-known effects in diabetes and hyperlipidemic disorders, pharmacological agents that target PPARs may have therapeutic applications in inflammatory diseases and cancer.

PPAR*α* was the first PPAR identified [10]. It has a wide tissue distribution being expressed in liver, skeletal muscle, intestine, kidney, adipose tissue, and vascular endothelial cells, tissues in which fatty acids are predominantly catabolized [11, 12]. Several ligands have been shown to bind PPAR*α* and to regulate transcriptional activity of target genes involved in the regulation of fatty acid metabolism as fatty acid transporters, catabolic enzymes involved in mitochondrial, and peroxisomal oxidation as well as genes necessary for the maintenance of redox balance during the oxidative catabolism of fatty acids [4, 5, 13]. PPAR*α*-activating ligands include a number of pharmacological compounds as well as fatty acid and fatty acid-derived molecules (reviewed in [2]). Lipid-lowering fibrates as Bezafibrate, Clofibrate, Fenofibrate and Gemfibrozil as well as certain nonsteroidal anti-inflammatory drugs (NSAIDs) bind and activate PPAR*α* [14, 15]. In addition, a variety of unsaturated and saturated fatty acids including arachidonic acid, palmitic acid, linoleic acid, linolenic acid, and oleic acid can act as natural ligands of PPAR*α* as they can bind and activate this receptor. Some eicosanoids derived from the metabolism of arachidonic acid such as leukotrienes (LTs), hydroxyeicosatetraenoic acids (HETEs), and prostaglandins (PGs) can also be effective ligand agonists for specific PPAR isoforms [16–19]. However, it is not clear whether the concentrations at which binding of these compounds occurs are physiologically relevant.

In addition to its known role in the regulation of genes involved in fatty acid metabolism, this isoform has been shown to attenuate the inflammatory response [6, 7, 9]. Most recently, an antitumoral role of PPAR*α* and its ligands has been proposed [7, 8]. This assumption is supported by recent experimental evidence, revealing antitumoral and anti-angiogenic properties of PPAR*α* activators both in vitro and in vivo.

## 2. ANTI-INFLAMMATORY ACTIONS OF PPAR*α*

In recent years, considerable attention has focused on the involvement of PPARs in inflammatory processes. Anti-inflammatory effects of PPAR ligands, in particular those of PPAR*α* and PPAR*γ*, have been ascribed to inhibition of the expression of inflammatory genes and negative interference with pro inflammatory transcription factor signaling pathways in vascular and inflammatory cells (reviewed in [1, 6, 20]. The first evidence for the involvement of PPAR*α* in the control of inflammation came from the studies of the inflammatory response elicited by LTB4 in PPAR*α* deficient mice. In these animals, inflammatory response to LTB4 was prolonged compared to WT mice, correlating with the ability of LTB4 to activate PPAR*α* and regulate the expression of genes involved in its own catabolism [21]. Thus, proinflammatory lipid metabolites may serve as ligands for PPARs thereby activating PPAR*α* responsive enzymes responsible for their clearance. Therefore, some of the anti-inflammatory actions of these receptors can occur through this autoregulatory loop in lipid homeostasis. Several lipids mediators, as the polyunsaturated fatty acids (PUFAs), can function as ligands for PPARs [2]. Arachidonic acid is a precursor of several eicosanoids that have pro inflammatory properties whereas the *ω*-3 PUFAs are precursors of anti-inflammatory eicosanoids as EPA (eicosapentanoid acid) and docosahexaenoic acid (DHA). These *ω*-3 PUFAs derivatives have been reported to decrease the production of several pro inflammatory cytokines, having beneficial effects in several inflammatory diseases as rheumatoid arthritis and inflammatory bowel disease [22, 23].

Several studies have confirmed the anti-inflammatory properties of PPARs in vitro and in vivo through the regulation of genes involved in the inflammatory response. PPAR agonists decrease plasma concentrations of interleukin (IL)-6, tumor necrosis factor (TNF)-*α*, and interferon (IFN)-*γ* in humans [24, 25]. In vascular smooth-muscle cells, PPAR*α* ligands inhibit IL-1-induced production of IL-6, COX-2, and prostaglandins [25]. PPAR*α* activation inhibits cytokine-induced expression of vascular cell-adhesion molecule-1 (VCAM-1) and vascular endothelial growth factor receptor-2 (VEGFR-2) in endothelial cells [26, 27]. PPAR*α* have been also involved in the downregulation of the production of IL-2 and TNF*α* by T lymphocytes [28].

**Figure 1:**
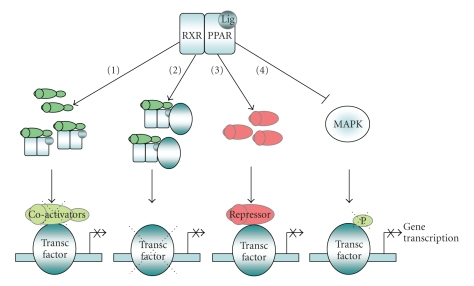
*Negative regulation of gene expression by PPARs.* Different mechanisms of transrepression through interference of activated PPARs with activation of transcription factors have been described. (1) Activated PPAR-RXR complexes may sequester essential coactivators shared with other transcription factors. (2) Physical association of PPARs with specific transcription factors results in reduced binding to their cognate response elements. (3) PPAR*α* agonists can also influence transcriptional activation by upregulating the expression of transcriptional repressors such as I*κ*B*α*. (4) PPAR*α*-mediated interference on the activation of members of the mitogen-activated protein kinase (MAPK) cascade influences transcription factor activation.

At the molecular level, most of the inhibitory actions of PPAR*α* on gene transcription result from its ability to transrepress the activities of many activated transcription factors such as nuclear factor (NF)-*κ*B [25, 29], activator protein-1 (AP-1) [29, 30] C/EBP*β* [31, 32], signal transducers and activators of transcription (STATs) [33], and transcription factor specificity protein 1 (Sp-1) [27]. Negative regulation of gene expression by PPAR*α* can occur by several mechanisms (Figure 1). Activated PPAR-RXR complexes may compete for limited amounts of essential coactivators shared with other transcription factors. Direct physical interactions between PPARs and specific transcription factors have been also proposed to mediate transcriptional inhibition by activated PPAR*α*, resulting in reduced binding to their cognate response elements. Agonists-activated PPAR*α* can effectively antagonize NF-*κ*B and AP-1-mediated signaling pathways in a bidirectional manner by physical interaction with the Rel homology domain of NF*κ*B-p65 and with the aminoterminal of c-Jun, respectively [34]. Physical association of the C-terminal DNA binding region of c/EBP*β* with PPAR*α* mediates inhibition of alpha1-acid glycoprotein gene expression [31]. PPAR*α* agonists can also influence transcriptional activation by modulating the expression of transcriptional repressors such as I*κ*B*α* (Kleemann, 2003 *#*1223; Vanden Berghe, 2003 *#*2170}). Finally, an additional mechanism of transrepression relies on the ability of some PPAR*α* ligands to interfere with the activation of certain members of the mitogen-activated protein kinase (MAPK) cascade as Jun kinase (JNK) and p38 MAPK [30, 35, 36].

## 3. INVOLVEMENT OF PPAR*α* IN CANCER

Rapidly accumulating evidence links members of the PPAR family and their agonists to cellular growth and tumor progression. The role of the PPAR*γ* isotype in cancer has been widely studied with a large number of reports demonstrating antitumoral properties of PPAR*γ* agonists in a variety of different malignancies [8]. Concerning to the involvement of the PPAR*α* form and its ligands in cancer, both tumor promotion and suppression properties have been reported [37]. Sustained PPAR*α* activation by agonists as clofibrate and Wy-14643 induce hepatocarcinogenesis in rodents [38, 39]. However, epidemiological data on long-term administration of PPAR*α* activators in the clinic discard the occurrence of these effects in human [40–43]. Discrepancies on the effects of PPAR*α* ligands in rodents and human liver seem to be due to several differences between species [44]. PPAR*α* mediated signaling is less efficient in human than in mice [37, 43, 45] and expression of this receptor is 10 to 20 times higher in rodent hepatocytes compared to human liver [46].

Emerging evidence indicates that PPAR*α* ligands are able to suppress the growth of different types of human carcinomas. PPAR*α* is expressed in a variety of human and murine tumor cell lines [47–49]. Expression of PPAR*α* have been also reported in clinical samples of several types of human cancers as colorectal carcinoma [50], prostate adenocarcinoma, [47, 51], testicular cancer [52], bladder carcinoma [53], and medulloblastoma [54]. PPAR*α* ligands are able to arrest the growth of human cancer cell lines in vitro and to slow the growth of transplanted human tumor cells in nude mice. These anticancer effects have been observed in several different cancer cell types including hepatoma [55], melanoma [56], glioblastoma and fibrosarcoma [49], endometrial and ovarian cancer [57–59], as well as colon carcinoma [50, 60, 61]. Clofibric acid, a ligand for PPAR*α* inhibits growth of ovarian cancer both in vivo and in vitro [58, 59]. Mice treated topically with the PPAR*α* ligand Wy-14643 exhibited diminished skin tumorigenesis [62]. Moreover, Fenofibrate suppresses the metastatic potential of melanoma cells [56, 63].

**Figure 2:**
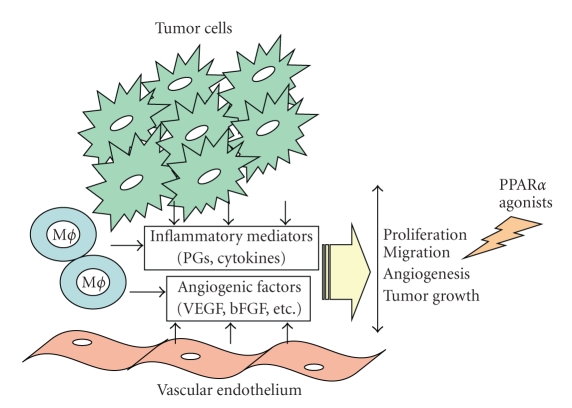
*Antitumoral effects of PPAR*α* ligands*. Tumor progression depends on a cascade of cellular signals involving: (a) proinflammatory factors (cytokines, COX-2 derived prostaglandins (PGs), chemokines); (b) proangiogenic factors (VEGF, bFGF, and PGs) produced by endothelial and inflammatory cells, stromal fibroblasts, and tumor cells. These factors promote cell proliferation, migration, and induce new vessels that deliver nutrients and oxygen to the malignant cells and therefore allow tumor growth and metastasis. PPAR*α* ligands may display antitumoral properties by their inhibitory effects on the transcription of genes involved in inflammation, cell growth, and angiogenesis thus leading to the inhibition of tumor growth.

Even though accumulative evidence shows data suggesting that PPAR*α* ligands may display antitumoral properties, the precise mechanism remains unclear. A number of reports suggest that antitumor properties of PPAR*α* activators reside on their anti-inflammatory and anti-angiogenic effects [27, 64, 65] (Figure 2). The dependence on the presence of PPAR*α* for the antitumorigenic and anti-angiogenic role of PPAR ligands has been determined by the analysis of their effects in PPAR*α* null mice. Wy-14643-mediated antitumoral and anti-angiogenic responses on tumor and endothelial cells are absent in PPAR*α* KO mice [66]. Panigrahy and coworkers [49] have shown the importance of the microenvironment in tumor progression in such a way that the activation of PPAR*α* expressed in endothelial and inflammatory cells of the host rather than in the tumoral cells is critical for anti-inflammatory, antitumor and anti-angiogenic activity of PPAR*α* agonists. Consistent with the anti-inflammatory role of this receptor, PPAR*α* null mice exhibit an increase of inflammatory infiltrates in tumors. Paradoxically, in spite of the enhanced inflammatory response in the absence of PPAR*α*, tumor growth is suppressed in these animals as a consequence of an increased production of anti-angiogenic factors TSP-1 and endostatin [67]. The immune system can have a multitude of effects on cancer development and progression, both favorable and detrimental [68, 69]. This apparent contradiction has been explained by the severity and duration of the inflammatory response associated to tumor growth. While acute inflammation is part of the defense response that may participate in the remission of preclinical cancers, chronic inflammation can promote tumor development with infiltrating innate immune cells providing proinflammatory and proangiogenic factors including cytokines, chemokines, VEGF, and prostanoids [70–72]. The association between cancer and inflammation has been also illustrated by epidemiological studies showing that the use of anti-inflammatory compounds in chronic inflammatory diseases reduces cancer risk tumor [73, 74]. In this sense, PPAR*α*-mediated anti-inflammatory actions can be responsible for their potential chemopreventive effects in tumor progression.

An emerging area of interest is the association of anti-inflammatory actions of dietary PUFAs as potential natural agonists of PPARs with cancer risk. Increasing evidence suggests that dietary fat regulation of gene expression can play a critical role in initiation and progression of human cancer and epidemiological studies have suggested an association between dietary fat and cancer risk [75, 76]. A number of reports have shown the beneficial effects of consumption of *ω*-3 PUFAs, associated with anti-inflammatory effects and with protection against primary tumor development [22, 23, 75]. Although some of the effects of dietary lipids can be linked to PPARs-mediated signaling, additional research is needed to understand the potential connection between dietary fat intake and PPARs in the control of inflammation and tumor progression.

## 4. PPAR*α*, COX-2, AND CANCER

### 4.1. COX-2: an essential role in inflammation and tumor growth

Cyclooxygenases (COX-1 and COX-2) convert arachidonic acid to prostaglandin H_2_(PGH_2_), an endoperoxide intermediate that, via specific synthases, is then transformed to prostaglandins (PGE_2_, PGD_2_, PGF_2*α*_, PGI_2_) and thromboxanes (TXA_2_). Whereas COX-1 is constitutively expressed in most tissues, COX-2 is transcriptionally upregulated in response to mitogens, tumor promoters, and pro inflammatory stimuli in a discrete number of cell types (reviewed in [77–79]). These enzymes are the target of nonsteroidal anti-inflammatory drugs (NSAIDs), one of the most widely used therapeutic for the relief of pain and inflammation (reviewed in [80–82]). The anti-inflammatory and analgesic efficacy of drugs arises essentially from inhibition of the enzymatic activity of COXs. As COX-2 is though to be the predominant isoform involved in the inflammatory response, the ability of NSAIDs to inhibit COX-2 activity may explain their therapeutic effects as anti-inflammatory drugs. Therefore, most of the new research on anti-inflammatory drugs has been aiming at targeting the COX-2 inducible production of PGs.

In addition to its essential role in inflammation, accumulating evidence links COX-2 with cancer and angiogenesis, suggesting that drugs that target COX-2 and related signaling cascades could be used as antitumoral agents [74]. Many human cancers display elevated COX-2 expression and studies in COX-2 null mice have demonstrated the role of this enzyme in tumor progression and metastasis [83–85]. Moreover, epidemiological studies have revealed a role of selective COX-2 inhibitors in decreasing the risk of developing colon cancer and in suppressing tumor formation and growth in animal models [73, 86–88]. COX-2 inhibitors can block both the production of angiogenic factors and the migration of vascular endothelial cells, and thus decrease tumor growth [89, 90]. Although some of the effects of these drugs on tumor regression might result from modulation of COX-2-independent pathways [91], it is likely that COX-2 is an important mediator of tumor growth.

### 4.2. Effects of PPAR*α* ligands on COX-2 expression

The activity of COX-2 is closely linked to PPARs as COX-2 catalyzes the production of fatty acid derivates as prostanoids that are PPAR activators [2]. Modulation of COX-2 activity should influence the local availability of PPAR ligands; therefore, indirectly modulating PPAR activity. Moreover, some NSAIDs may act as PPAR*α* and *γ* ligands, suggesting that, in addition to inhibit prostaglandin production, they might regulate gene expression as part of their anti-inflammatory and chemopreventive mechanisms [92, 93]. Downregulation of COX-2 expression by PPAR*α* ligands may account for some of the anti-inflammatory, anti-angiogenic and antitumoral properties of these drugs in a variety of cell types [20] (Table 1). PPAR*α* agonists as Wy-14643 inhibit macrophage differentiation and COX-2 gene expression [94]. In liver, COX-2 upregulation upon experimental nutritional steatohepatitis is suppressed by the PPAR*α* agonist Wy-14,643 in *wt* but not in PPAR*α* KO mice. This effect has been ascribed to the ability of activated PPAR*α* to interfere with the NF-*κ*B-signaling pathway [95]. Transcriptional interference of activated PPAR*α* with NF-*κ*B also explains the inhibition of COX-2 induction and PG production in response to IL-1 in vascular smooth muscle cells [25]. The NF-*κ*B target genes VCAM-1 and COX-2 are also downregulated by PPAR*α* ligands in response to cytokine activation [29]. Regarding to experimental support for anti-angiogenic and antitumoral actions of these drugs related to their effects on COX-2 expression, it has been shown that Fenofibrate inhibits bFGF-mediated angiogenesis and COX-2 mRNA expression in endothelial cells [65]. Panigrahy and cols have observed suppression of COX-2 expression in Fenofibrate and Wy-14643 treated tumors [49]. Clofibric acid suppresses the growth of tumor xenograts of ovarian cancer cell lines with decreased microvessel density, PGE_2_ production, and COX-2 and mPGES-1 expression [58, 59]. Diminished expression of COX-2 upon PPAR*α* agonist treatment was parallel with reduced expression of AP-1. Similarly, in colon carcinoma cells, PPAR*α* agonists severely diminish phorbol ester-mediated AP-1-dependent induction of COX-2 expression [30].

## 5. PPAR*α*, VEGF, AND CANCER

### 5.1. VEGF: a therapeutic target in tumoral angiogenesis

Angiogenesis, defined as the formation of new blood vessels from preexisting vasculature, occurs under physiological conditions during embryonic development and is required for wound healing and reproduction in the adult. Indispensable for physiological processes, angiogenesis is highly regulated via fine tuning of the balance between pro- and anti-angiogenic factors [97]. Excessive angiogenesis is tightly linked to human disease, including chronic inflammatory disease, diabetic retinopathy, and cancer [98, 99]. Ample evidence shows that blockade of tumoral angiogenesis often relieves the severity of cancer [100].

Both cancer cells and cells attracted to the sites of inflammation are able to produce proangiogenic factors that cause endothelial cell recruitment and proliferation for the supply of oxygen and nutrients that favor the growth of solid tumors and facilitate metastasis. In this context, tumor-associated hypoxia plays an essential role in the regulation of angiogenesis [101]. Response to hypoxia is mediated by members of the hypoxia-inducible transcription factors (HIFs) involved in the regulation of the expression of genes participating in oxygen homeostasis [102, 103]. In addition, hypoxia has been also found to drive induction of potent angiogenic and inflammatory factors including VEGF, VEGF-R1 and -R2, angiotensin, metalloproteinases, and COX-2 [104–106]. This response of tumors to hypoxia contributes to the malignant phenotype and to more aggressive tumor progression [107].

It has been well established that VEGF signaling pathway is one of the key regulators in angiogenesis [108, 109]. Cytokines, growth factors, tumor promoters, and hypoxia modulate the expression of VEGF [110, 111]. Activated VEGF may bind to two types of tyrosine kinases receptors: VEGFR-1 and VEGFR-2. Proangiogenic actions of VEGF as vascular endothelial cell permeability, proliferation migration, and survival are mediated mainly through binding an activation of the VEGFR-2 [112, 113]. Accumulating evidence supports a key role of VEGF in cancer, contributing to tumor neovascularization and dissemination. Increased expression of this factor has been found in most tumors, and agents neutralizing VEGF expression or activity inhibit tumor growth in vivo (reviewed in [99, 100, 114]).

### 5.2. Inhibition of VEGF signaling by PPAR*α* agonists

PPAR*α* ligands can inhibit endothelial cell proliferation and migration and induce endothelial cell apoptosis in vitro, suggesting an important role of this receptor in tumor angiogenesis. Part of these effects occurs through the ability of PPAR*α* ligands to interfere with VEGF-mediated signaling. (Table 2). At the transcriptional level, PPAR agonists have been shown to inhibit endothelial VEGFR-2 expression by repressing transactivation and binding of Sp1 to DNA [27]. Wy-14643 downregulates TPA-mediated transcriptional induction of VEGF by interference with activation of AP-1 [30]. Interestingly, lipid-lowering therapy with Fenofibrate induces a significant reduction of VEGF levels in serum [96]. Anti-angiogenic actions of PPAR*α* agonists may explain some of their antitumoral effects. Fenofibrate reduces adventitial angiogenesis and inflammation in a porcine model of coronary angioplasty [115]. Both Fenofibrate and Wy-14643 are able to suppress VEGF secretion in glioblastoma cells and Lewis lung carcinoma cells and to inhibit angiogenesis both in vivo and in vitro [49]. Moreover, Clofibric acid inhibits VEGF expression in tumor xenografts of ovarian cancer cell lines with a reduction in angiogenesis and decreased microvessel density in solid tumors [58, 59].

## 6. CONCLUSIONS

Taken together, findings on the effects of PPAR*α* ligands in inflammation and cancer, suggest that PPAR*α* activation may be beneficial against tumorigenesis through the inhibition of transcriptional activation of genes involved in inflammation and angiogenesis such as COX-2 and VEGF. Although COX-2 and VEGF are one of the many proinflammatory and proangiogenic factors that drive tumor growth and metastasis, targeting these proteins suffices to significantly impair tumor growth and angiogenesis. Inhibition of tumor inflammation and tumor angiogenesis by PPAR*α* ligands might be responsible for their potential chemopreventive effects in a variety of experimental models of cancer. However, it must be taken into account that many of the reported effects of PPAR*α* ligands on tumor progression have been obtained in vitro and await confirmation by additional basic and clinical research to ascertain whether they can be considered of pharmacological significance in vivo.

## Figures and Tables

**Table 1:** Effects of PPAR*α* ligands on COX-2 signaling.

PPAR*α* ligand	Action/effect	References
Wy-14643		
	Inhibition of IL-1-induced COX-2 expression in vascular smooth muscle cells	[25]
	Inhibition of LPS-induced COX-2 expression in THP-1 monocytes	[94]
	Inhibition of TPA-induced COX-2 expression in colon carcinoma cell lines	[30]
	Inhibition of COX-2 expression in B16-F10 melanoma tumor	[49]
	Inhibition of COX-2 up regulation by experimental steatohepatitis in liver	[95]

Fenofibrate		
	Inhibition of COX-2 expression in B16-F10 melanoma tumor	[49]
	Inhibition of b-FGF induced COX-2 expression in endothelial cells	[65]

Clofibric acid		
	Inhibition of COX-2 expression in tumor xenografts	[58]
	Inhibition of mPGES-1 expression in tumor xenografts	[59]

**Table 2:** Effects of PPAR*α* ligands on VEGF signaling.

PPAR*α* ligand	Action/effect	References
Wy-14643		
	Inhibition of VEGF-mediated endothelial cell migration	[27, 49, 64]
	Inhibition of VEGF production by glioblastoma U87 cells	[49]
	Inhibition of VEGF-induced phosphorylation of Akt	[64]
	Inhibition of VEGF-mediated angiogenesis in vitro	[27]

Fenofibrate		
	Inhibition of VEGF production by glioblastoma U87 cells	[49]
	Reduction of plasma VEGF	[96]
	Inhibition of VEGF-induced phosphorylation of Akt	[64]
	Inhibition of VEGFR2 expression in endothelial cells	[27]

Clofibric acid		
	Inhibition of VEGF expression in tumor xenografts	[58]
